# Ectopic Expression of *WUS* in Hypocotyl Promotes Cell Division via *GRP23* in *Arabidopsis*

**DOI:** 10.1371/journal.pone.0075773

**Published:** 2013-09-26

**Authors:** Dajian Zhang, Xiaomin Wang, Min Wang, Junhua Li, Xiaoyu Guo, Kang Chong, Yunyuan Xu

**Affiliations:** 1 Key Laboratory of Plant Molecular Physiology, Institute of Botany, Chinese Academy of Sciences, Beijing, China; 2 University of Chinese Academy of Sciences, Beijing, China; Universidad Miguel Hernández de Elche, Spain

## Abstract

WUSCHEL (WUS) is essential for preventing stem cell differentiation in *Arabidopsis* . Here we report that in addition to its functions in meristematic stem cell maintenance, WUS is involved in the regulation of cell division. The *WUS* gain-of-function mutant, stem ectopic flowers (sef), displayed elongated hypocotyls, whereas the loss-of-function *wus-1* mutant had shortened hypocotyls. The long hypocotyl in *sef* was due to the presence of more cells, rather than increased cell elongation. Microscopic observation, flow cytometry assays, quantitative RT-PCR (qRT-PCR), and histochemical staining of *CycB1;1::GUS* supported the hypothesis that ectopic cell division occurred in the *sef* hypocotyls after germination. Both immunoblot and qRT-PCR results showed that *WUS* was ectopically expressed in *sef* hypocotyls. Luciferase activity, chromatin immunoprecipitation (ChIP) and electrophoretic mobility shift assay (EMSA) showed that *GLUTAMINE-RICH* PROTEIN *23* (*GRP23*) expression can be activated by WUS and that *GRP23* is a direct target gene of WUS. The phenotypes of *35S::GRP23* plants and *GRP23* knockdown lines supported the notion that *GRP23* mediates the effects of *WUS* on hypocotyl length. Together, our data suggest that ectopic expression of *WUS* in hypocotyl controls cell division through its target gene *GRP23*.

## Introduction

Stem cells in meristems maintain proliferation potential and continuously produce new cells that cease cell division, exit the meristem, and take on specific growth patterns in response to environmental, developmental and hormonal cues [1]. In shoot meristems, the WUSCHEL (WUS) transcription factor is sufficient to prevent stem cell differentiation [2,3], and *wus-1* mutants have disorganized and premature termination of shoot meristems [4]. Stem cell maintenance depends in part on a negative feedback loop mediated by *WUS* and *CLAVATA 3* (*CLV3*) [3]. WUS directly represses the expression of several A-type members in the *ARABIDOPSIS THALIANA RESPONSE REGULATOR* (*ARR*) gene family, which are negative regulators of cytokinin signaling [5,6]. Previous research has revealed that there is a positive feedback loop between *WUS* and the cytokinin signaling pathway [7-10]. In this loop, WUS activates cytokinin signaling by repressing A-type *ARRs*; in turn, cytokinin promotes *WUS* expression via ARABIDOPSIS HISTIDINE KINASE 4 (AHK4), which is a cytokinin receptor [9,11]. The antagonistic activities of cytokinin and CLV3 restrict *WUS* expression to three to four cells [5].

As a transcription factor, WUS directly binds to at least two distinct DNA motifs found in more than 100 target promoters [12]. It preferentially affects the expression of genes with roles in hormone signaling, metabolism, and development. *GLUTAMINE-RICH* PROTEIN *23* (*GRP23*) is one of the genes directly targeted by WUS [12]. The interaction between GRP23 and RNA polymerase II functions in transcriptional regulation for early embryogenesis in *Arabidopsis* [13]. These findings suggest a possible link between WUS and *GRP23* in embryogenesis.

Hypocotyl length is affected by both cell number and cell elongation. Cell number is fixed during embryogenesis in wild type, and no further cell division occurs during hypocotyl growth [14]. Thus, differences in hypocotyl length depend mainly on the elongation of each cell, which is tightly controlled by environmental factors such as light and hormones including auxin, Gibberellic Acid (GA) and Brassinosteroid (BR) [15-17]. Dark-grown dicotyledonous plants have longer hypocotyl cells compared to light-grown ones [18].

We have reported the phenotypes of *WUS* gain-of-function mutant identified via activation tagging genetic screening. The mutant exhibits clustered ectopic floral buds on the surface of inflorescence stems. The mutant is designated as *sef* for *stem ectopic flowers*. Our previous observation indicated that the ectopic floral meristems are initiated from the differentiated cortex cells [19]. In this study, characterization of mutants revealed that *WUS* functions in cell division in hypocotyl. In *sef*, *WUS* is ectopically expressed in hypocotyl where WUS directly binds to the *GRP23* promoter to activate its expression. The expression of *GRP23* caused extra cell division, which ultimately leads to aberrantly long *sef* hypocotyls.

## Results

### Hypocotyls of *sef* are longer than those of wild type

**Figure 1:**
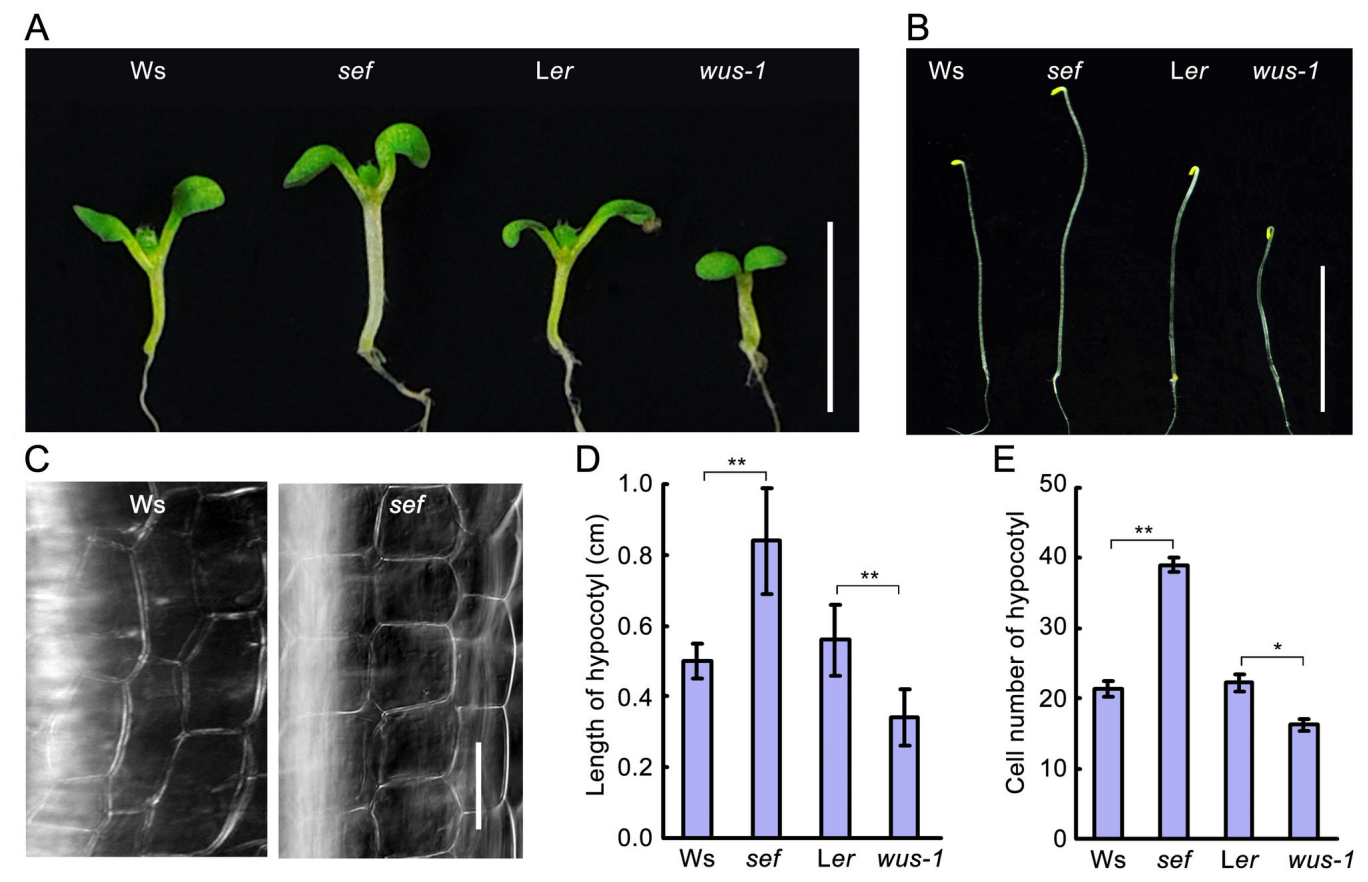
Hypocotyl phenotypes of *sef* and *wus-1*. (A) Hypocotyl phenotype of *WUS* gain-of-function (sef) and loss-of-function (wus-1) mutant seedlings grown in 16-h light/8-h dark. Bar = 1 cm. (B) Hypocotyl length of dark-grown seedlings of *sef* and *wus-1* mutant and their corresponding wild type. Bar = 1 cm. (C) Comparison of the cell number in a same length of hypocotyl in 8-day-old seedlings. Bar = 50 µm. (D) Hypocotyl length of 8-day-old seedlings. Data are means ± SD (*n* > 15) Student’s *t* test, **P < 0.01. (E) Hypocotyl cell number of 8-day-old seedlings. Data are means ± SD (*n* > 15). Student’s *t* test, **P < 0.01, *P < 0.05.

*sef* is a gain-of-function mutant in which endogenous *WUS* expression is dramatically elevated; the mutant exhibits clustered ectopic ﬂoral buds on the surface of inﬂorescence stems [19]. Here, we further examined *sef*, finding that it had elongated hypocotyls compared to wild type Ws. This was the case in both light-grown and dark-grown seedlings (Figure 1A and 1B). Under light conditions, the hypocotyls in *sef* were about twice as long as those of Ws. By contrast, hypocotyls in the *wus-1* loss-of-function mutant were about third shorter than those of wild type L*er* (Figure 1D). To investigate the reason underlying the elongated hypocotyl phenotype in *sef*, we examined the number of epidermal cells in 8-day-old seedlings (Figure 1C and 1E). The *sef* hypocotyls contained about twice as many cells as those of Ws, whereas *wus-1* contained fewer than L*er*. The differences of hypocotyl length and cell number in hypocotyl are significant between wild type and mutant (*P* < 0.05). These results indicate that *sef* and *wus-1* mutants have aberrant hypocotyl lengths due to altered hypocotyl cell production.

### The cell division rate is increased in *sef* hypocotyls

**Figure 2:**
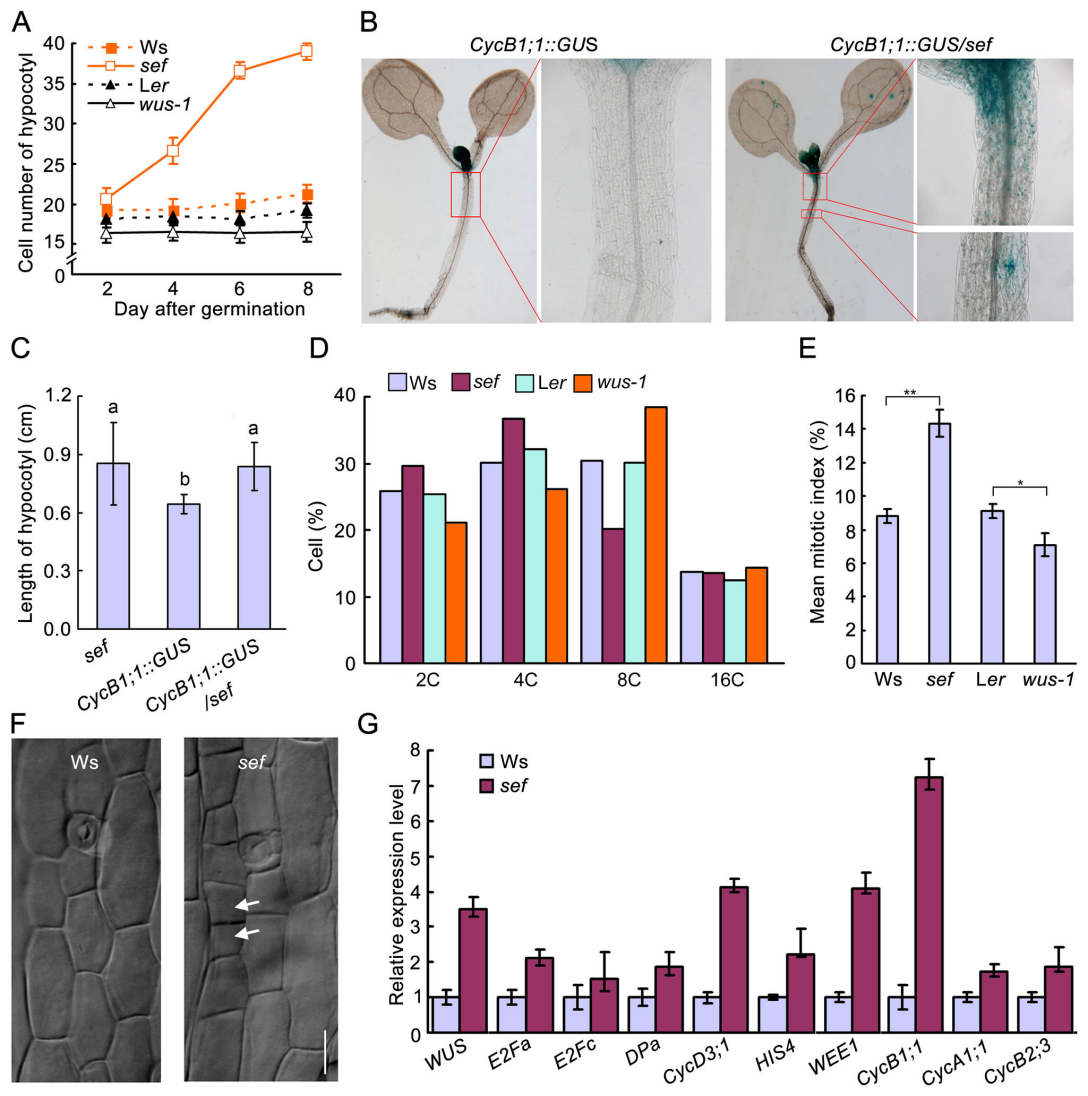
Aberrant cell division in hypocotyl of *sef*. (A) Cell number of hypocotyl at given days after germination. Data are means ± SD (*n* > 15). (B) *CycB1;1::GUS* expression patterns in 8-day-old seedling of *CycB1;1::GUS* and *CycB1;1::GUS*/*sef*. (C) Hypocotyl length of 8-day-old *sef*, *CycB1;1::GUS* and *CycB1;1::GUS/sef* seedlings. Data shown are average values ± SD (*n* > 15). Different letters represent significant differences according to Student’s *t* test, *P < 0.05. (D) Cell cycle progression in hypocotyls of Ws, *sef*, L*er* and *wus-1* detected by flow cytometry. (E) Mean mitotic index in hypocotyls of Ws, *sef*, L*er* and *wus-1*. Student’s *t* test, **P < 0.01, *P < 0.05. (F) Cell division in the hypocotyls of 4-day-old *sef* seedlings. Bar = 25 µm. (G) Expression levels of cell cycle-related genes in wild type and *sef*. Data are means ± SD (n = 3).

To investigate cell accumulation in the hypocotyl, we monitored cell numbers at different times after germination. Our results showed that cells in the hypocotyl of *sef* divided faster than those of the wild type at 2, 4 and 6 days after germination (Figure 2A). By contrast, cells in *wus-1* and wild-type hypocotyls almost don’t divide during 2- to 8-day after germination. These results suggest that enhanced expression of *WUS* promotes cell division in the hypocotyl after germination.

*CycB1;1::GUS* is a classic marker used to investigate cell division [20]. We generated Ws and *sef* plants harboring *CycB1;1::GUS*. Strong GUS activity was detected in young leaves and the shoot apical meristem of *CycB1;1::GUS* seedlings (Figure 2B). In *CycB1;1::GUS*/*sef* seedlings, GUS activity was additionally observed in the hypocotyls (Figure 2B). The hypocotyls of *CycB1;1::GUS*/*sef* seedlings were longer than those of *CycB1;1::GUS* seedlings, similar to those of *sef* compared to wild type Ws (Figure 1D and Figure 2C).

To investigate the effect of WUS on cell cycle progression, we measured ploidy levels of hypocotyl cells by flow cytometry. The numbers of 2C and 4C cells were significantly higher in *sef* than in Ws. In *wus-1*, there were fewer of both 2C and 4C cells than in wild type L*er*. There were fewer 8C cells in *sef* than in Ws, and more in *wus-1* than in L*er*. A high fraction of 2C and 4C cells and a low fraction of 8C cells can be indicative of promotion of mitosis [21]. As such, our results suggest that more cells with 2C and 4C in the G2/M phase in *sef* hypocotyls but less in *wus-1* hypocotyls (Figure 2D).

The mitotic index is defined as the ratio of the number of cells in mitosis to the total number of cells and is used as an indicator of the proliferation status in a cell population [21]. The mitotic index in the hypocotyls of *sef* and wild type was calculated based on the flow cytometric assay. In *sef* hypocotyls, the mitotic index was significantly higher than in Ws (*P* < 0.01 by Student’s *t* test) (Figure 2E). Consistent with this, cell division could be observed in the hypocotyl epidermis of 4-day-old *sef* seedlings (Figure 2F). This suggests that cytokinesis took place in the hypocotyl of *sef*.

Expression levels of checkpoint-related genes in cell cycle were analyzed by quantitative RT-PCR (qRT-PCR). The tested genes included: G1-S transition genes *E2Fa*, *E2Fc*, *DPa* and *CycD3; 1*; S phase gene *HIS4*; and G2-M transition genes *WEE1*, *CycB1; 1*, *CycB2;3*, and *CycA1; 1* [21,22]. Our results demonstrated that in *sef*, *E2Fa* and *DPa* expression increased 2-fold, and that of *CycD3; 1* increased by more than 4-fold compared to wild type. We also found that expression of the S phase gene *HIS4* was increased about 3-fold in *sef* compared to wild type. The expression levels of both *WEE1* and *CycB1; 1* were up regulated more than 4-fold in *sef* (Figure 2G). These qRT-PCR results showing increased expression of cell cycle-related genes are consistent with cell division taking place in the *sef* hypocotyl.

We also examined seed and cotyledon size in *sef*. Compared to wild type, *sef* seeds and cotyledons were dramatically larger (Figure S1A, S1B and S1C). The size of the palisade cells in *sef* was similar to that of the Ws (Figure S1E). However, there were more cotyledon cells in *sef* than in Ws (Figure S1D). These results further confirm that *sef* has a higher cell division rate than wild type, leading to larger cotyledons as well as longer hypocotyls.

### *WUS* is expressed ectopically in *sef* hypocotyls

**Figure 3:**
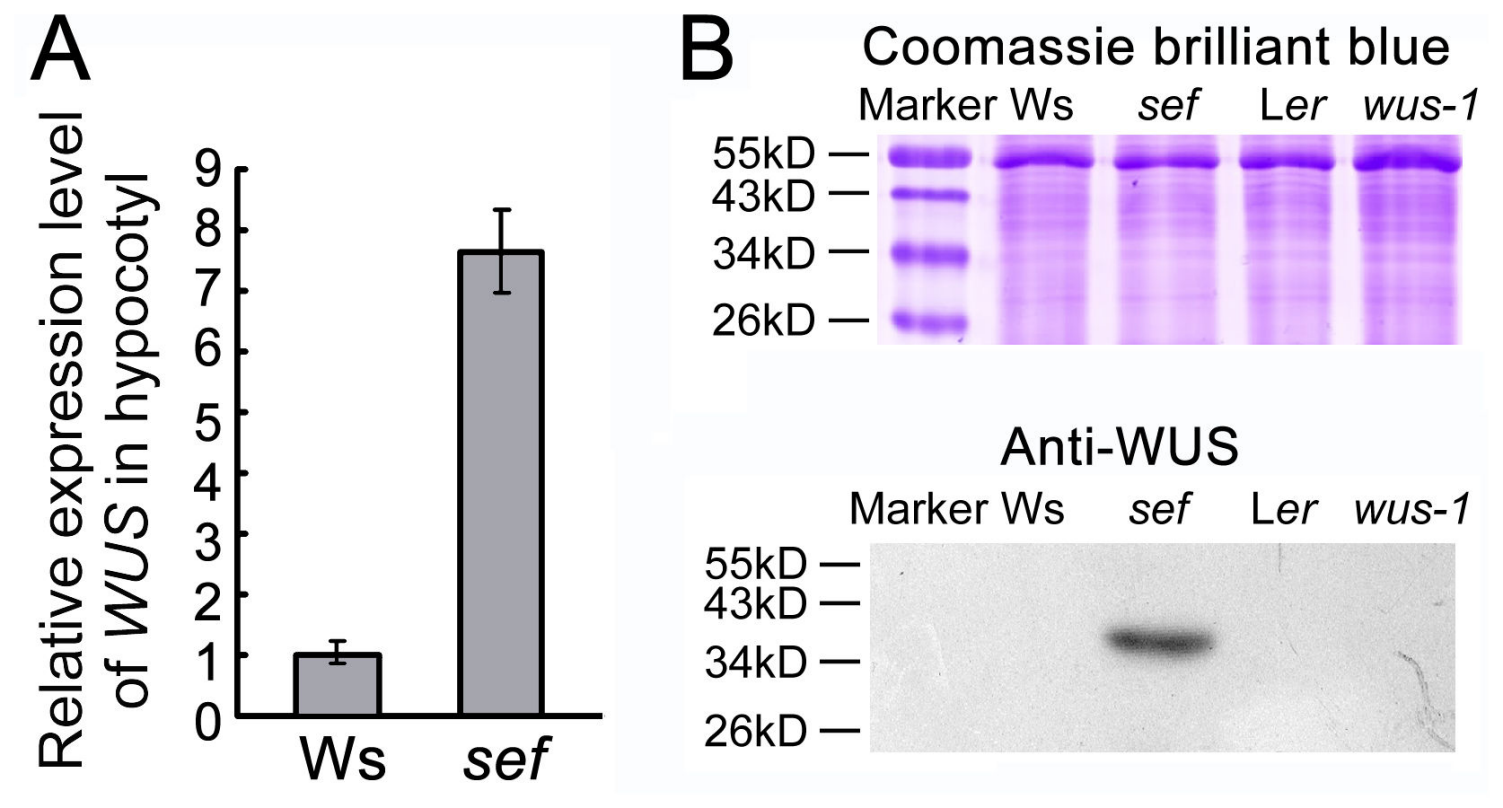
*WUS* is expressed ectopically in hypocotyls of *sef*. (A) Expression of *WUS* in hypocotyls of Ws, *sef*, L*er* and *wus-1* detected by qRT-PCR. Data are means ± SD (n = 3). (B) Immunoblot analysis of WUS protein in hypocotyls of Ws, *sef*, L*er* and *wus-1*. Upper panel, coomassie brilliant blue (CBB)-stained SDS-PAGE gel. Bottom panel, immunoblotting of WUS protein.

Based on the increased cell division rate in the hypocotyl of *sef* and the reduced rate in *wus-1*, we hypothesized that the increased *WUS* levels might be responsible for the extra cell division in *sef*. To investigate this, RT-PCR was used to examine the expression of *WUS* in hypocotyls. Total RNA was isolated from the hypocotyls of 8-day-old Ws and *sef* seedlings. *WUS* transcript was detected after 25 cycles in the *sef* hypocotyl samples, but not in the wild-type samples. At 40 cycles, the amplification of *WUS* was saturated in *sef*, but the transcripts was still undetectable in the wild-type hypocotyls (Figure S2A). We also used qRT-PCR to check the transcriptional level of *WUS* (Figure 3A), and immunoblotting to examine the WUS protein level (Figure 3B) in the *sef* hypocotyls. Our results showed that both the RNA and protein of *WUS* were detected in the *sef* hypocotyls but not in the wild type (Figure 3A and 3B). These data demonstrate that, unlike in wild type, *WUS* is expressed in *sef* hypocotyls.

### WUS binds the GRP23 promoter directly to activate its expression

WUS directly binds to at least two distinct DNA motifs in the promoters of its target genes, the TAAT motif [23] and TCACGTGA [12]. WUS has been reported to have more than 100 direct targets, including genes involved in development, hormone signaling, and cell division. Based on the presence of these motifs in its promoter, *GRP23* is one of the potential direct targets of WUS [12]. Our qRT-PCR analysis revealed that *GRP23* expresses not only in flower and root but also in hypocotyl in wild type (Figure S3).

**Figure 4:**
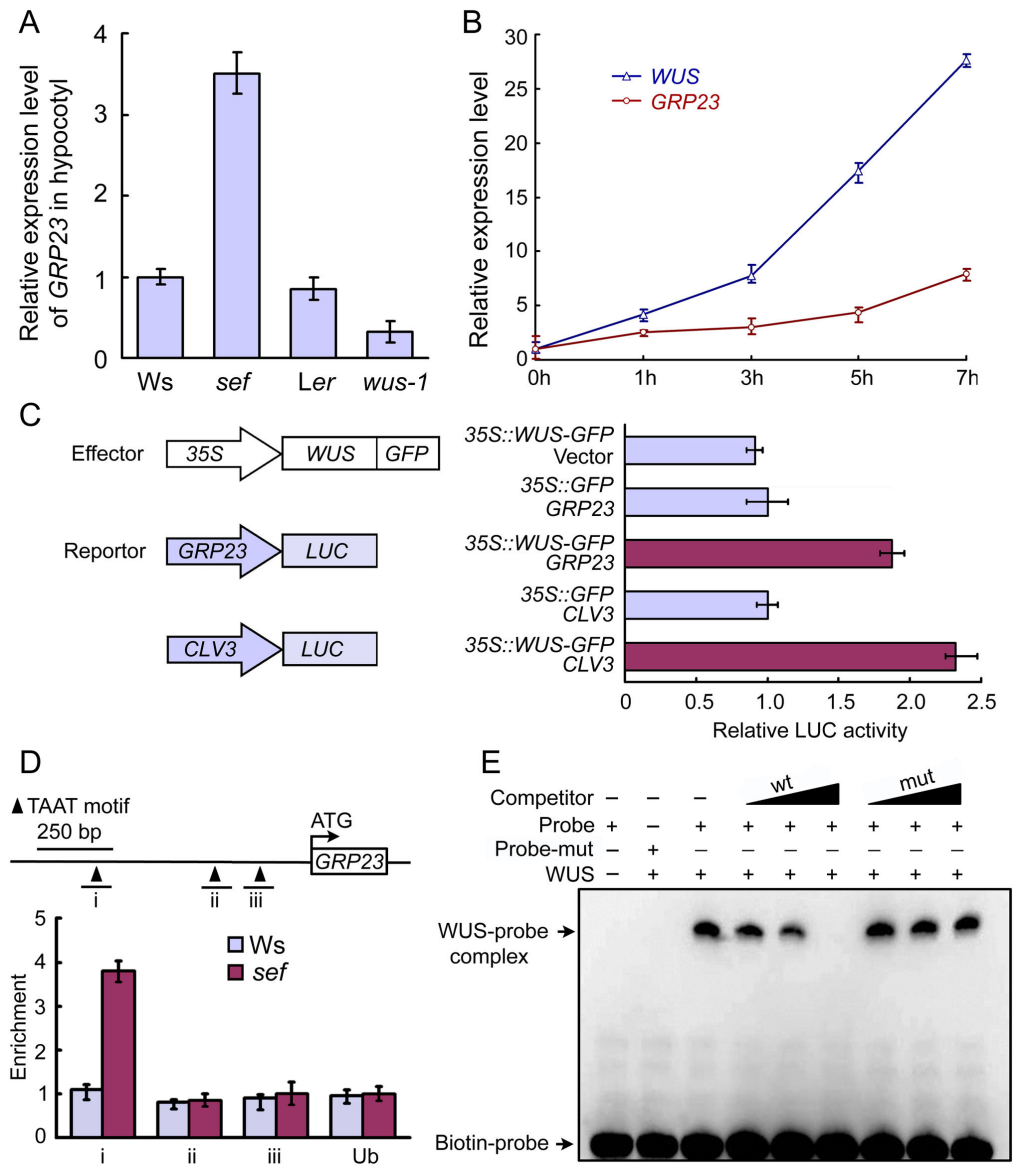
WUS binds the *GRP23* promoter directly to activate its expression. (A) qRT-PCR analysis of *GRP23* expression in Ws, *sef*, L*er* and *wus-1*. Data are means ± SD (n = 3). (B) qRT-PCR analysis of *GRP23* expression in 14-day-old *pga6* seedlings after inducing with 17-β-estradiol for 1, 3, 5 and 7 hours. Data are means ± SD (n = 3). (C) Transient expression assay in *Arabidopsis* protoplasts. The promoters of *GRP23* and *CLV3* were used to drive the *luciferase* (LUC) reporter gene. *WUS:GFP* fusion driven by the 35S promoter was used as effector. LUC activity was assayed after transformation. Data are means ± SD (n = 3). (D) ChIP assay of 7-day-old seedlings to show WUS-binding regions in *GRP23* promoter. Regions i, -784 to -660; ii, -311 to -211; iii, -195 to -95. Data are means ± SD (n = 3). (E) EMSA of WUS binding the *GRP23* promoter *in* *vitro*. The unlabeled double-strands probe (wt) and unlabeled mutant probe (mut) were used for competitive inhibition with 200X, 400X, or 800X molar excess.

To address the relationship between WUS and *GRP23*, the expression levels of *GRP23* in *sef* and *wus-1* hypocotyls were examined by qRT-PCR. *GRP23* transcripts were 3.5-fold more abundant in *sef* compared to the Ws, whereas in *wus-1*, *GRP23* expression was 0.32-fold that of the L*er* (Figure 4A). *GRP23* expression was also monitored in *pga6-1*, an inducible *WUS* overexpression line [24], after *WUS* expression was induced with 17-β-estradiol for different lengths of time. The expression of *GRP23* increased upon induction of *WUS* expression in *pga6* (Figure 4B).

To test whether WUS directly binds to the promoter of *GRP23*, we performed transient expression assays, chromatin immunoprecipitation (ChIP) assays, and electrophoretic mobility shift assays (EMSAs). We performed transient activation assays using protoplasts from *Arabidopsis* . The *LUCIFERASE* (*LUC*) gene driven by the *GRP23* promoter (2.0 kb upstream of ATG) was transformed along with various effector constructs into *Arabidopsis* protoplasts. The promoter of *CLV3*, a target gene of WUS [25], was used as a positive control. When *Arabidopsis* protoplasts were co-transfected with the reporter plasmids containing *GRP23::LUC* or *CLV3::LUC* and the effector plasmid containing *35S::WUS*, the relative LUC activity was increased by 1.9- and 2.4-fold compared to the control (Figure 4C). Thus, our results indicate that WUS serves as an activator for *GRP23* transcription in protoplasts.

To further determine whether WUS directly associates with the promoter sequence of *GRP23 in vivo*, we performed ChIP assay. As shown in Figure 4D, the region “i” of the *GRP23* promoter, which included two TAAT motifs from -784 to -660, were enriched with higher abundance in *sef* compared with Ws.

In EMSA experiments using biotin-labeled fragments with 40 base pairs of *GRP23* promoter (-748 to -709), covering two TAAT motifs, a clear WUS-dependent mobility shift was identified (Figure 4E). The unlabeled fragments competitively inhibited this binding. When the TAAT motif was replaced by GGGG, the unlabeled mutated fragments cannot influence the binding of WUS protein (Figure 4E). It is indicated that WUS proteins directly bind to the promoter region of *GRP23 in vitro*. Taken together, these data suggest that *GRP23* expression can be activated by WUS directly in *sef* hypocotyl.

### The expression of *GRP23* affects hypocotyl length

**Figure 5:**
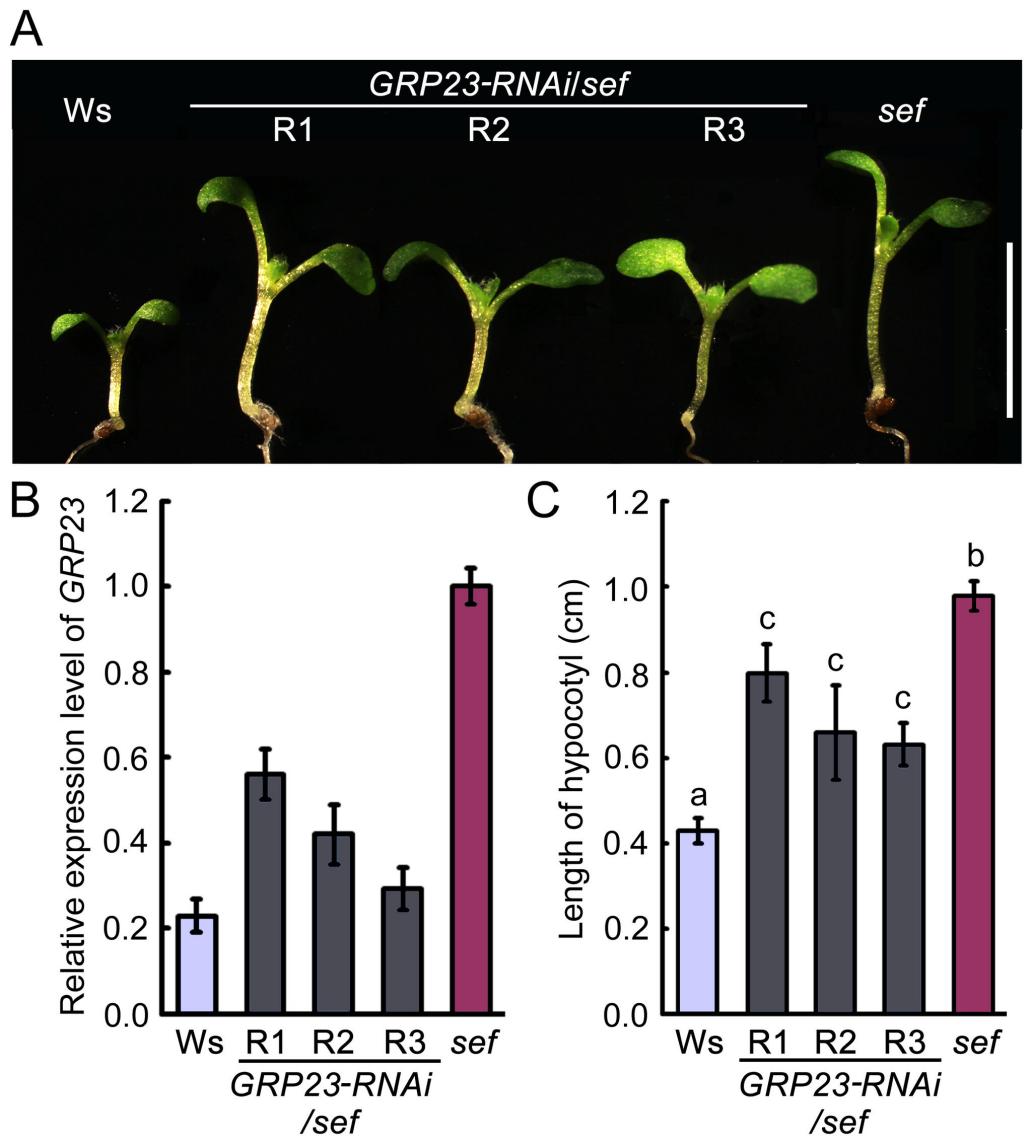
Knockdown of *GRP23* partially rescues the *sef* phenotype. (A) Hypocotyl phenotype of Ws, *sef* and *sef* *GRP23-RNAi* seedlings after germination for 8 days. Bar = 1 cm. R1, R2, and R3 represent the RNAi lines 1, 2, and 3 respectively. (B) Expression of *GRP23* in *sef* and *GRP23-RNAi/sef* seedlings detected by qRT-PCR. Data are means ± SD (n = 3). (C) Hypocotyl length of 8-day-old seedlings of Ws, *sef* and *GRP23-RNAi/sef*. Data are means ± SD (*n* > 15). Different letters a, b, and c represent significantly differences among the lines (*P < 0.05) by Student’s *t* test.

To test the role of *GRP23* in hypocotyl growth, we used an RNAi approach to generate three independent *GRP23* knockdown transgenic lines in the *sef* background (*GRP23-RNAi/sef*). qRT-PCR analysis revealed a reduction of *GRP23* transcript to 58%, 44%, and 27% in the three transgenic lines R1, R2, and R3, respectively (Figure 5B and Figure S2B). Hypocotyl length in the transgenic lines was intermediate between those of Ws and *sef* (Figure 5A and 5C). These results demonstrate that knockdown of *GRP23* can partially attenuate the elongated hypocotyl phenotype of *sef*.

**Figure 6:**
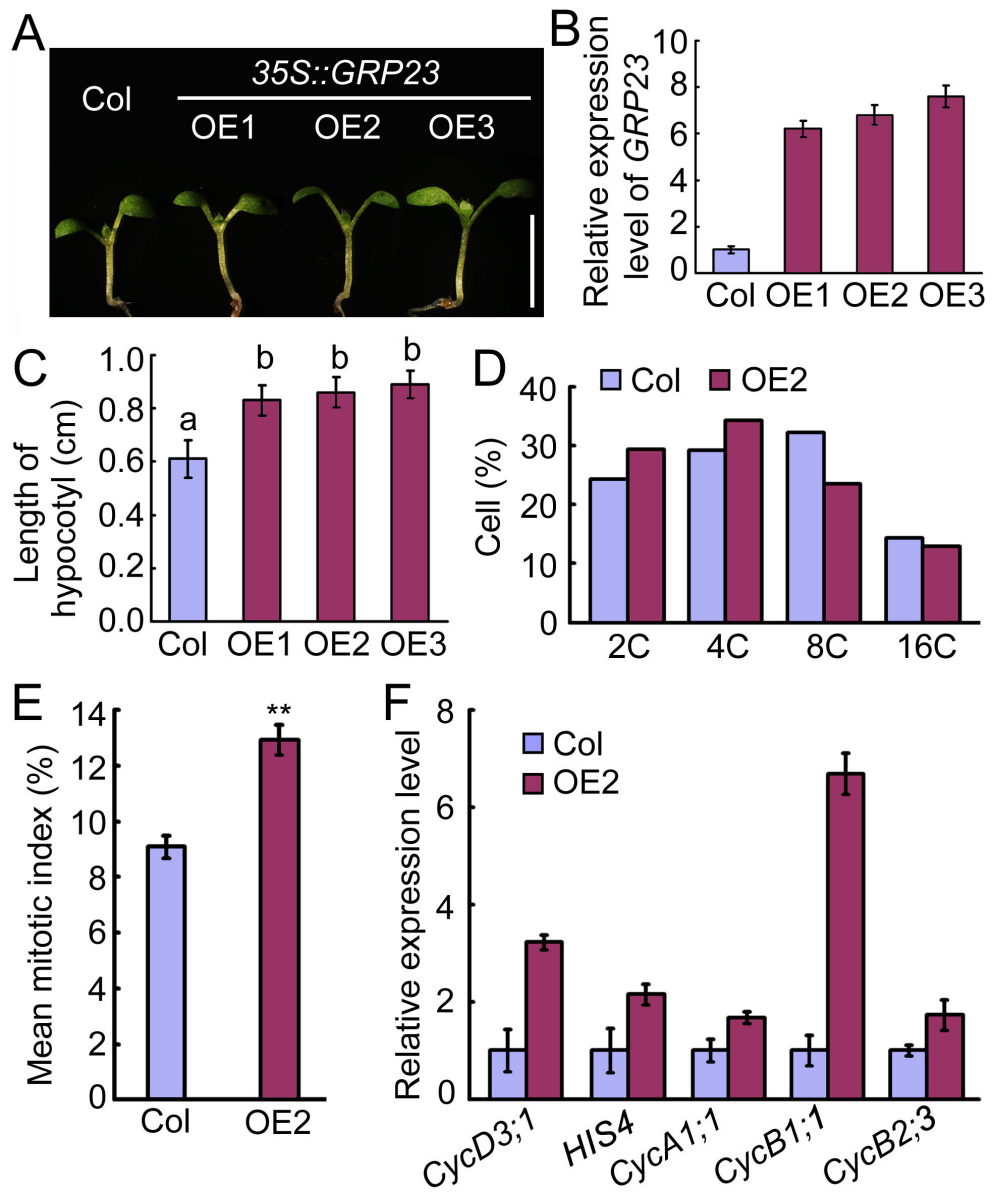
Phenotype of *35S::GRP23* transgenic plants. (A) Hypocotyl phenotype of Col and *35S::GRP23* transgenic lines (OE1, OE2, and OE3) after germination for 8 days. Bars = 1 cm.(B) GRP23 expression in Col and *35S::GRP23* seedlings detected by qRT-PCR. Data are means ± SD (n = 3). (C) Hypocotyl length of Col and *35S::GRP23* seedlings after germination for 8 days. Data are means ± SD (*n* > 15). Different letters a and b represent significantly differences among the lines (*P < 0.05) by Student’s *t* test. (D) Cell cycle progression in hypocotyls of Col and *35S::GRP23* line 2 (OE2) analyzed by flow cytometry. (E) Mean mitotic index in hypocotyl of Col and *35S::GRP23* line 2. Student’s *t* test, **P < 0.01. (F) Expression levels of cell cycle-related genes in Col and *35S::GRP23* line 2. Data are means ± SD (n = 3).

To confirm the function of *GRP23* in hypocotyl cell division, three independent *35S::GRP23* transgenic lines, OE1, OE2, and OE3 were obtained. *GRP23* transcript was markedly increased in all three lines compared with wild type (Figure 6B). In addition, hypocotyl length in the three *35S::GRP23* lines was significantly increased compared to wild type (*P* < 0.05) (Figure 6A and 6C). These results indicate that *GRP23* overexpression can mimic the elongated hypocotyl phenotype of *sef*.

We also performed flow cytometry assays to examine the cell cycle in hypocotyls of OE2. The hypocotyls of *35S::GRP23* plants possessed more cells with 2C or 4C in the G2/M phase and had a higher mitotic index than those of the wild type (Figure 6D and 6E). The expression levels of cell cycle checkpoint-related genes were elevated in *35S::GRP23* hypocotyls compared to those of wild type (Figure 6F). These data suggest that *GRP23* promotes cell division in the hypocotyl through controlling the G2/M transition.

## Discussion

### Aberrantly long hypocotyls in *sef* are caused by ectopic expression of *WUS*

WUS specifies stem cell identity in the cells overlying of the central zone, and is both necessary and sufficient for stem cell maintenance [2,4]. Moreover, *WUS* is connected with *CLV3* through a regulatory loop for maintaining a constant number of stem cells [2,26,27]. Previous studies have mainly concentrated on the mechanism through which *WUS* maintains the number of stem cells in the shoot and floral meristems. Here we report the effect of ectopic WUS on cell division in hypocotyl.

Cell number in the hypocotyl is constant, with approximately 20 cells in *Arabidopsis* [14,16]. Although a considerable number of mutants with altered hypocotyl length have been studied, these reports all focused on cell elongation [15,28]. For example, *Arabidopsis* *ENHANCED PHOTOMORPHOGENIC 1* (*EPP1*) encodes an ATP-dependent chromatin remodeling factor. EPP1 interacts with HY5 to regulate cell elongation in the hypocotyl [29]. MICROTUBULE-DESTABILIZING PROTEIN 25 (MDP25) modulates hypocotyl length by affecting cell elongation [30]. By contrast, our results revealed that the long and short hypocotyls in *sef* and *wus-1*, respectively, were caused not by differential cell elongation, but by the presence of a different number of cells (Figure 1). In addition, more cells were also found in seeds and cotyledons of *sef* (Figure S1). *WUS* is normally expressed in cells of the organizing center and in overlying cells of the central zone [2,4]. We speculated that *WUS* might be ectopically expressed in *sef* hypocotyls based on the previously established ectopic *WUS* expression in the inflorescence stem of *sef* [19]. Indeed, we found evidences of *WUS* expression at both the transcript and protein level in hypocotyls of the *sef* mutant (Figure 3). It is likely that the ectopic *WUS* expression in *sef* is due to insertion of 35S enhancers in the *WUS* promoter [19]. Other previous reports also showed a similar phenomenon, in wild-type *Arabidopsis* , no transcripts of the *HOMEODOMAIN GLABROUS 11* (*HDG11*) gene can be detected in roots, leaves, or stems, but 35S enhancers resulted in the overexprssion of *HDG11* in a constitutive fashion [31]. Together, these results indicate that *WUS* is involved in controlling hypocotyl length in *sef* by altering cell number. The increase of cell number in *sef* hypocotyls resulted from the activation of *GRP23* by ectopic WUS.

WUS directly represses the transcription of *ARR5*, *ARR6* and *ARR7*, which act as negative regulators of cytokinin signaling [6]. The expression of *ARR5*, *ARR6* and *ARR7* was inhibited in *sef* hypocotyls (Figure S4). Based on these results, it is likely that ectopic expression of *WUS* in *sef* hypocotyls results in an enhanced cytokinin signal to activate cell division.

### WUS regulates *GRP23* to mediate cell division in the hypocotyl

Post embryonic growth of *Arabidopsis* hypocotyl, cell division in the hypocotyl occurs only in the epidermis during the formation of stomata. The elongation of hypocotyls does not involve cell division in the cortex or epidermis [14,16,32]. However, our results showed that the increase in cell number of *sef* hypocotyls occurred mainly during postembryonic development (Figure 2A). Reporter gene expression levels and flow cytometry assay results also supported the idea that cell division occurred in *sef* hypocotyl due to *WUS* ectopic expression (Figures 2, 3). Ectopic expression of *WUS* driven by the 35S promoter occasionally causes activation of the *CycB1;1::GUS* reporter gene along the vasculature of leaves [33]. These results indicate that WUS can promote cell division in tissues outside of the organizing center of stem cells in *Arabidopsis* .

The pathway through which WUS activates cell division is unknown. However ChIP-chip results revealed that *GRP23* is one of the 159 direct WUS target genes and can be induced by WUS in *Arabidopsis* apices [12]. Moreover, the histochemical assay of *GRP23::GUS* and *in situ* hybridization showed that *GRP23* expresses in the embryo, ovules, primordium of leaf and lateral root, and apical meristems of root and shoot [13]. The expression patterns of *GRP23* and *WUS* overlap in embryo and shoot meristem [2,3,13]. Based on these results it can be speculated that *GRP23* is a direct target of WUS in the wild-type meristem. Our ChIP, EMSA and LUC activity results showed that WUS directly binds the *GRP23* promoter to activate reporter gene expression (Figure 4). Our results are consistent with the previous findings of Busch et al. [12]. Together, these results indicate that WUS directly targets *GRP23* to activate its expression both in wild-type shoot apical meristem and in *sef* hypocotyl.

Reduced expression of *GRP23* rescued the elongated hypocotyl phenotype of *sef*, whereas *GRP23* overexpression resulted in a higher mitotic index and increased expression of cell division related genes, mimicking *sef* (Figure 5 and Figure 6). These results support the idea that WUS promotes cell division via *GRP23*, which encodes a PENTATRICOPEPTIDE REPEAT (PPR) protein. The *grp23* mutant displays an aberrant cell division pattern [13]. Mutants of another PPR protein gene, *PPR2263*, exhibit growth defects and reduced size resulting from altered cell division [34]. These reports are consistent with our observation that the PPR protein GRP23 promotes cell division.

In conclusion, our data suggest that ectopic expression of *WUS* in hypocotyl regulates cell division via promoting *GRP23* expression. *GRP23* is a direct target gene of transcription factor WUS that mediates it effects on cell division in hypocotyls.

## Materials and Methods

### Plant materials and growth conditions

The gain-of-function mutant *sef* (ecotype Ws-2) was identified via activation tagged genetic screening as described previously [19]. *wus-1* (ecotype L*er*) was obtained from the *Arabidopsis* Biological Resource Center at Ohio State University (Columbus, USA). The *pga6-1* mutant and *35S::GRP23* transgenic plants were kindly provided by Prof. Jianru Zuo [24] and Prof. Weicai Yang [13] (Institute of Genetics and Developmental Biology, CAS, Beijing, China). Seeds were surface-sterilized with 10% bleach plus 0.01% Triton X-100 for 15 min, and then washed four times with sterile water. The surface-sterilized seeds were stratified at 4 °C for 2 days and transferred to medium or soil for further growth (16-h light/8-h dark, 22°C). *pga6-1* was treated with 10 µM 17-β-estradiol for different amounts of time as described by Zuo et al. [24].

### Measurement of length and cell number in hypocotyls

For phenotype analysis, seedlings were grown on 0.8% phytoagar plates containing half-strength Murashige-Skoog nutrients and 1% sucrose. Image J1.34 (http://rsb.info.nih.gov/ij/download.html) was used to measure hypocotyl length, seed size, and cotyledon size after photographing. Hypocotyl length was measured from the base of the cotyledon to the junction of the hypocotyl and the primary root.

To count the cell number, 2- to 8-day-old seedlings, were mounted with a clearing solution [35]. After 15–60 min, samples were examined under microscope (Lecia DM2500, Germany). The cell numbers in cotyledons and hypocotyls were counted. Quantitative data were subjected to two-tailed independent Student’s *t* tests using SPSS 18.0 software (http://www.spss.com). Significance levels of *P* < 0.05 and *P* < 0.01 are indicated by single and double asterisks, respectively.

### Flow cytometry analysis

For flow cytometry analysis, seedlings were plated onto half-strength Murashige-Skoog media. After 8 days in the greenhouse (16-h light/8-h dark, 22°C), hypocotyls were collected for flow cytometry analysis as previously described by Galbraith et al. [36]. The nuclei were analyzed with a ploidy analyzer FACS Caliber (BD Corporation). At least three biological replicates were used for each sample.

### RT-PCR and quantitative RT-PCR (qRT-PCR)

Total RNA was isolated from 8-day-old *Arabidopsis* hypocotyls or seedlings using TRIzol reagent (Invitrogen). The DNase-treated RNA was reverse-transcribed using M-MLV reverse transcriptase (Promega). cDNAs were synthesized from 2.0 µg total RNA using Superscript reverse transcriptase. RT-PCR was performed with gene-specific primers (Table 1) and runs 18-40 cycles depending on the linear range of products for each gene. RT-PCR reactions were repeated five times.

**Table 1:** Primer sequences.

**qRT-PCR**	
*WUS*-F	5'-GCTAATTCCGTCAACGTTAAAC-3’
*WUS*-R	5'-TTTAAATTCCCGTTATTGAAGC-3’
*WEE1*-F	5’-TTGGACAAAAGCTTACCAGTAGAAG-3’
*WEE1*-R	5’-AGAGAAGATATCGACTTTATCAAGG-3’
*HIS4*-F	5’-TTAGGCAAAGGAGGAGCAAA-3’
*HIS4*-R	5’-CTCCTCGCATGCTCAGTGTA-3’
*CycD3*;*1*-F	5’-GCAAGTTGATCCCTTTGACC-3’
*CycD3*;*1*-R	5’-CAGCTTGGACTGTTCAACGA-3’
*CycB1*;*1*-F	5’-CTCAAAATCCCACGCTTCTTGTGG-3’
*CycB1*;*1*-R	5’-CACGTCTACTACCTTTGGTTTCCC-3’
*CycA1*;*1*-F	5’-GGCTAAGAAGCGACCTGATG-3’
*CycA1*;*1*-R	5’-TACAAGCCACACCAAGCAAC-3’
*CycB2*;*3*-F	5’-TAAACCACCTGTGCATCGAC-3’
*CycB2*;*3*-R	5’-ATCTCCTCCAGCATTGCTTC-3’
*E2Fa*-F	5’-ACGCTGGTTCTCCTATCACAC-3’
*E2Fa*-R	5’-GGCTTGTTTAATTAGATTGACGAA-3’
*E2Fc*-F	5’-GGAAGGGTGCTGACAATCTT-3’
E2Fc-R	5’-CATCCAACCTGCTTTCCTCA-3’
*DPa*-F	5’-GATGATTCTGAAATTGGATCAGAG-3’
*DPa*-R	5’-TTGGCTTCCAACTTCTGACA-3’
*GRP23*-F	5’-TGCTCCATCCTCAGTTACTT-3’
*GRP23*-R	5’-AATAAACTCGCAGCATCTCC-3’
*ACTIN*-F	5’-GCTCCTCTTAACCCAAAGGC-3’
*ACTIN*-R	5’-CACACCATCACCAGAATCCAGC-3’
*ARR5*-F	5'-TTTGCGTCCCGAGATGTTAG-3’
*ARR5*-R	5'-CCATACTATCATCAACAGCAAGAAC-3’
*ARR6*-F	5’-TTGCCTCGTATTGATAGATGTC-3’
*ARR6*-R	5’-CGAGTGAACAGGGTAGACATT-3’
*ARR7*-F	5’-AATGCCAGGACTTTCAGGAT-3’
*ARR7*-R	5’-ATTCCTCTGCTCCTTCTTTG-3’
**RT-PCR**	
*WUS*-pBI221-F	5'-CCGCTCGAGATGGAGCCGCCACAGCATCAGCATC-3’
*WUS*-pBI221-R	5'-GGGGTACCCTAGTTCAGACGTAGCTCAAGAGAA-3’
*GRP23-LUC-*F	5’-CGGGATCCTATCCAGCTAATCCCATCTGCTCTT-3’
*GRP23-LUC*-R	5’-CCCAAGCTTGGTGGAGGGAAAATGATTTAGGGTT-3’
*CLV3-LUC-*F	5’-CGGGATCCGCAACCTTCGATAGAAATAGTGAC-3’
*CLV3-LUC*-R	5’-CCCAAGCTTAAGACACAAGTATATCTCCAAAGC-3’
*GRP23 RNAi-*F	5’-GGCGCGCCGGATCCCGGAGATGCTGCGAGTT-3’
*GRP23 RNAi-*R	5’-CATGCCATGGTCTAGACTTACTTCCGACCTTCTT-3’
*ACTIN*-F	5’-TTTGCGACAATGGAACTG-3’
*ACTIN*-R	5’-AAGAGCAATGTAGCAAAG-3’’
**EMSA**	
*GRP23p*-Probe-F	5'-CATATATCTTTAATACTGTTAATGATCTTTCTTCAAAAAC-3’
*GRP23p*-Probe-R	5'-GTTTTTGAAGAAAGATCATTAACAGTATTAAAGATATATG-3’
**ChIP**	
*GRP23p* i-F	5'-TCACGTTATATGAGCATCTTTT-3’
*GRP23p* i-R	5'-TTGAAACTGA AACTTTATACGAAA-3’
*GRP23p* ii-F	5'-ACCAGCTATGGATTATTTGAGA-3’
*GRP23p* ii-R	5'-AAGACAAGTAAAGAAAGGTTGG-3’
*GRP23p* iii-F	5'-CGTATTACCAAACAGCCCTC-3’
*GRP23p* iii-R	5'-CCTTGGATGTGAAGAAATGG-3’
*UBQ*-F	5'-CAGGATAAGGAGGGCATT-3’
*UBQ*-R	5'-TTTCCCAGTCAACGTCTT-3’

qRT-PCR was performed on an Applied Biosystems 7500 real time PCR System using SYBR Premix Ex Taq™ (TaKaRa). The following thermal cycle was used: 95°C for 3 min, then 40 cycles of 95°C for 30 s, 60°C for 30 s, and 72°C for 1 min. The *Actin1* gene (accession no. X16280) was used as the internal control. The relative expression levels were analyzed using a relative quantitation method (∆∆CT) for every PCR. The primers used for qRT-PCR are listed in Table 1.

### Immunoblotting

Total protein samples were extracted from 8-day-old *Arabidopsis* hypocotyls as described previously [37]. Proteinase inhibitors were added and proteins were separated on 12% SDS-PAGE gels and then transferred to a polyvinylidene ﬂuoride (BioTrace^TM^, USA) membrane. Membranes were blocked for 1 h with 5% BSA in TBS-Tween buffer (Tris-HCl 20 mM, NaCl 150 mM, and Tween 0.05%, pH 8.0). Immunoprobing of WUS was conducted with the rabbit anti-WUS (A gift from Huiqin Ma) (1:3,000) polyclonal antibody in TBS. An anti-rabbit IgG (1:10,000) conjugated with alkaline phosphatase was used as the secondary antibody with an ECL protein gel blot detection system (Amersham, Sweden).

### LUC activity assay

Protoplast isolation and transient expression assays were performed as described by Lin et al. [38]. *GRP23* and *CLV3* promoters (2kb) were amplified from genomic DNA and inserted into the reporter plasmid to drive the expression of *LUC*. To produce the effector plasmid, the full-length *WUS* CDS was inserted into the pBI221 plasmid and driven by CaMV 35S promoter. The primers used for amplification were listed in Table 1. For transient expression assays, the reporter plasmids pYY96-GRP23::LUC or pYY96-CLV3::LUC and effector constructs pBI221-WUS were cotransformed into protoplasts. The reporter gene *GUS* driven by 35S promoter was used as an internal control to normalize *LUC* expression. GUS fluorescence was detected with a UV fluorescence optical kit using a GLOMAX 20/20 LUMINOMETER (Promega). LUC activity was measured using LUC assay substrate with a luminescence kit (Promega). The relative reporter gene expression levels were expressed as the LUC/GUS ratios.

### Chromatin immunoprecipitation assays

Chromatin immunoprecipitation (ChIP) was performed as described with 7-day-old seedlings [39]. The rabbit anti-WUS polyclonal antibody was used for immunoprecipitation. ChIP products were analyzed by qRT-PCR, and the enriched relative abundance was expressed as the ratio of *sef* to Ws. Data are means ± SD of three independent experiments.

### EMSA

EMSA was performed essentially as described [40]. Briefly, the coding sequence of *WUS* was cloned into the expression vector pGEX-4T-1. The recombinant pGEX-4T-1-*WUS* was transformed into *Escherichia coli* BL21. Cells were grown at 37°C and induced by 1 mM isopropyl β-D-1-thiogalactopyranoside for 5 h and puriﬁed by glutathione afﬁnity chromatography as described in the Bulk and RediPack GST puriﬁcation kit (Pharmacia). EMSAs were performed using the biotin-labeled probes and the Lightshift Chemiluminescent EMSA kit (Pierce) according to the manufacturer’s instructions. Wild-type and mutated oligonucleotides were synthesized as single-stranded DNA. The wild-type oligonucleotide sequence with 40 bases corresponds to the -748 to -709 regions in the *GRP23* promoter. In the mutated oligonucleotide, two TAAT motifs (-738 to -735 and -729 to -726) were replaced by GGGG. Single-strand oligonucleotides were labeled with biotin at 3'-end, and then equal amounts of labeled complementary oligonucleotides were mixed, boiled for 2 min, and then slowly cooled down to 25°C for annealing. The labeled double-strand fragments were detected according to the instructions provided with the EMSA kit (Pierce). For competition experiments, different amounts of unlabeled wild-type and mutated double-strand fragments were added to the binding reaction.

## Supporting Information

Figure S1 **Seed and cotyledon phenotypes of *sef*.**
(DOCX)

Figure S2 **Expression levels of *WUS* and *GRP23* detected by RT-PCR.**
(DOCX)

Figure S3 **Expression levels of *GRP23* in various tissues of *Arabidopsis* *.***
(DOCX)

Figure S4 **Expression levels of A-type *ARRs* genes in hypocotyl.**
(DOCX)
