# Supplementary material for: Ectopic Expression of WUS in Hypocotyl Promotes Cell Division via GRP23 in Arabidopsis
Source: PLoS One. 2013 Sep 26;8(9):e75773. doi: 10.1371/journal.pone.0075773 (PMC3784395; doi:10.1371/journal.pone.0075773)
Supplement: Figure S1 — Seed and cotyledon phenotypes of sef. (DOCX) [file pone.0075773.s001.docx]

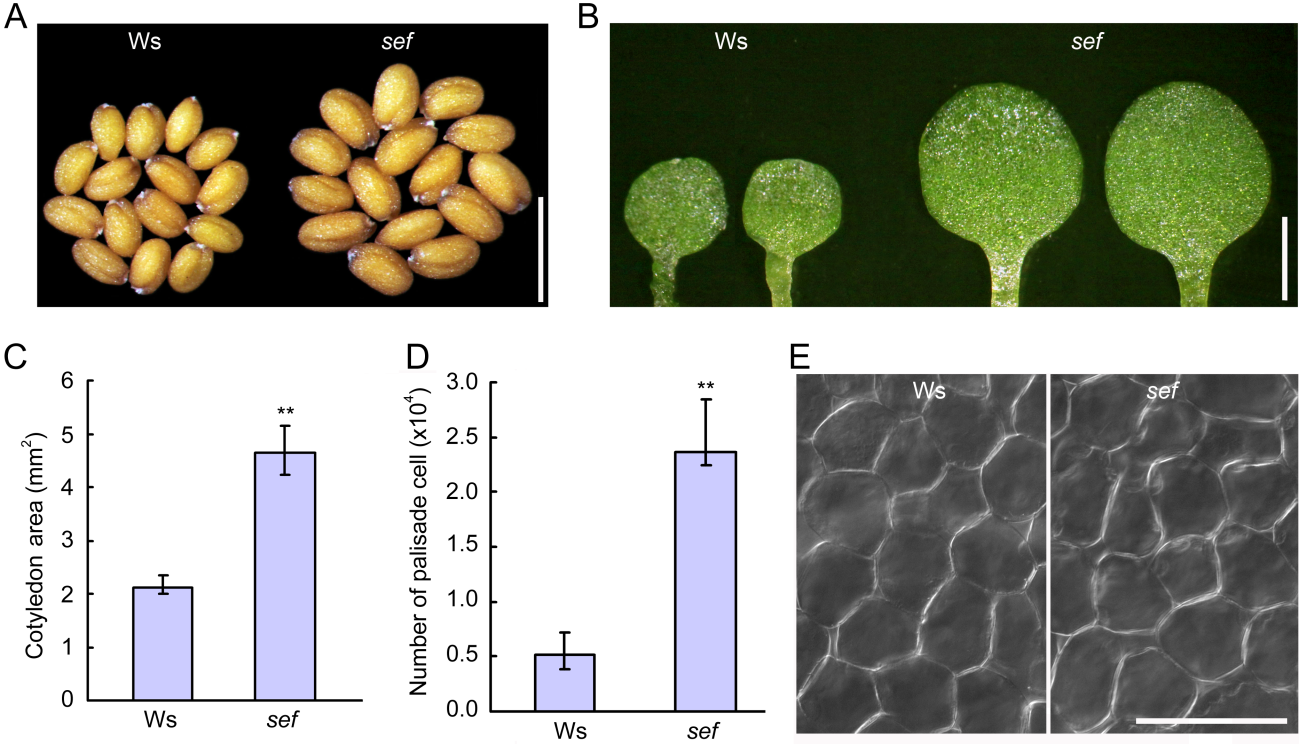


**Figure S1. Seed and cotyledon phenotypes of *sef***

(A) The *sef* seeds are larger than those of Ws*.* Bar = 2 mm. (B) Cotyledons in Ws and *sef.* Bar =1 mm. (C) Comparison of blade area of cotyledons of Ws and *sef*. Data are means ± SD (*n* > 15). Student’s *t* test, ***P* < 0.01. (D) Comparison of palisade cell number in cotyledons of Ws and *sef*. Data are means ± SD (*n* > 15). Student’s *t* test, ***P* < 0.01. (E) Palisade cells of cotyledons in Ws and *sef*. Bar = 50 μm.
