# Supplementary material for: Ectopic Expression of WUS in Hypocotyl Promotes Cell Division via GRP23 in Arabidopsis
Source: PLoS One. 2013 Sep 26;8(9):e75773. doi: 10.1371/journal.pone.0075773 (PMC3784395; doi:10.1371/journal.pone.0075773)
Supplement: Figure S2 — Expression levels of WUS and GRP23 detected by RT-PCR. (DOCX) [file pone.0075773.s002.docx]

**
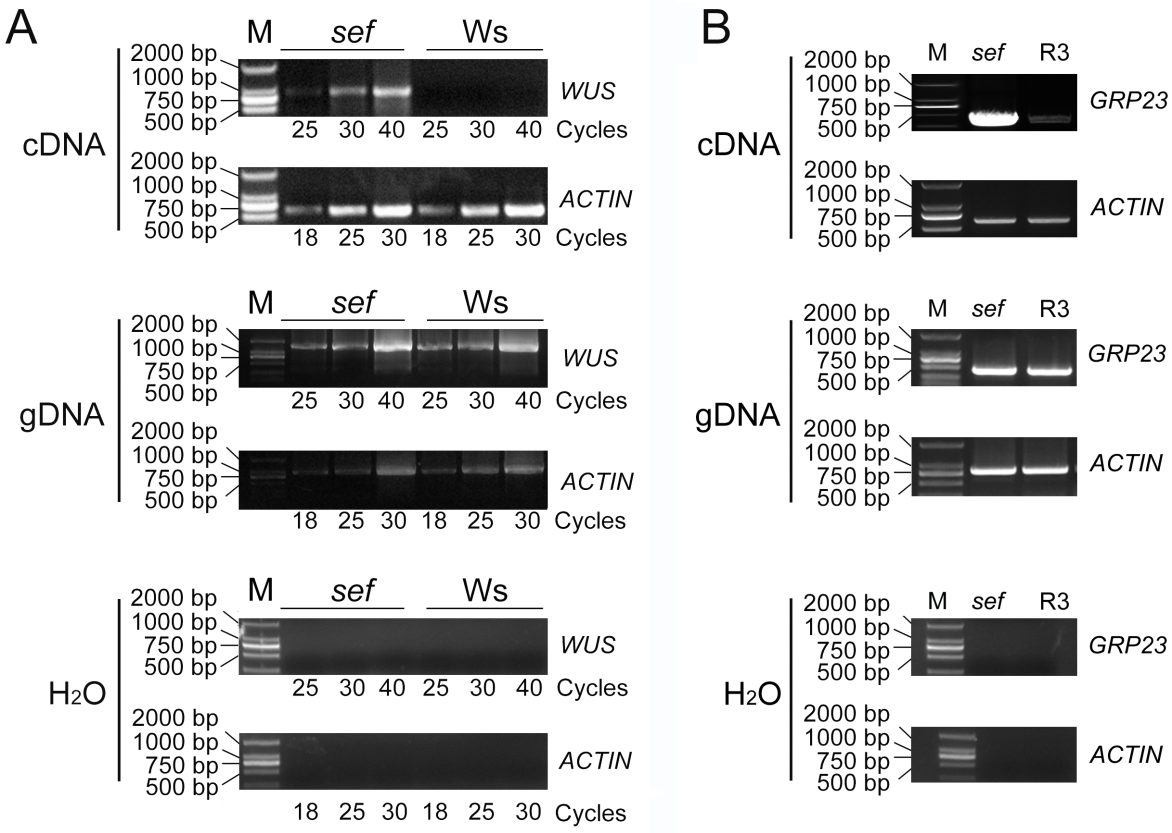
**

**Figure S2. Expression levels of *WUS* and *GRP23* detected by RT-PCR**

(A) *WUS* expression in hypocotyls of wild type and *sef*. Total RNA was isolated from hypocotyls of 8-day-old seedlings. *ACTIN* and genomic DNA (gDNA) were used as control. (B) Expression of *GRP23* in *sef* and *GRP23-RNAi/sef* line 3 (R3) seedlings detected by RT-PCR.
