# Supplementary material for: Ectopic Expression of WUS in Hypocotyl Promotes Cell Division via GRP23 in Arabidopsis
Source: PLoS One. 2013 Sep 26;8(9):e75773. doi: 10.1371/journal.pone.0075773 (PMC3784395; doi:10.1371/journal.pone.0075773)
Supplement: Figure S3 — Expression levels of GRP23 in various tissues of Arabidopsis . (DOCX) [file pone.0075773.s003.docx]

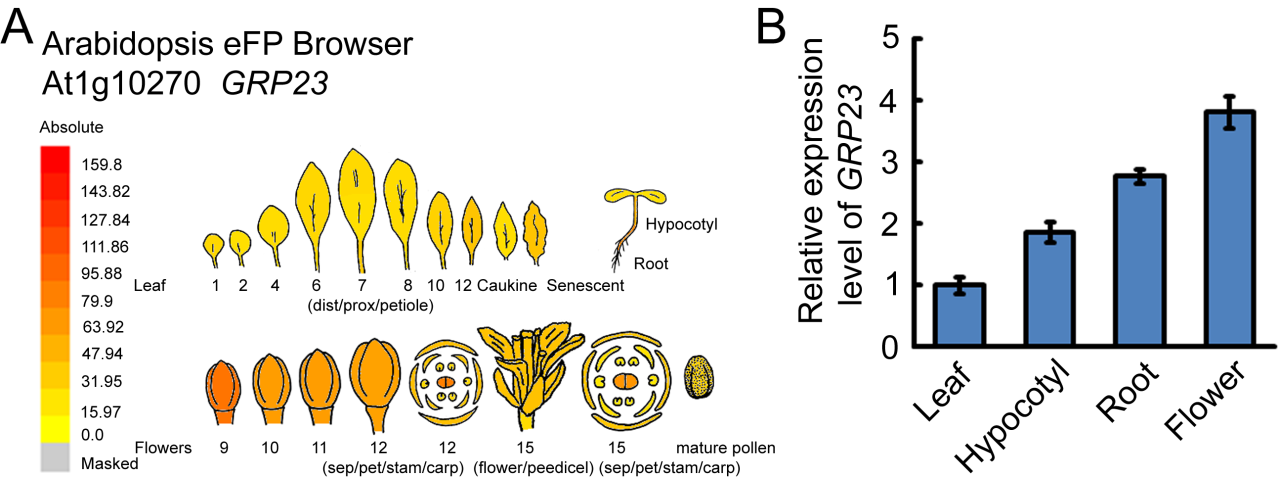


**Figure S3. Expression levels of *GRP23* in various tissues of *Arabidopsis***

(A) Relative expression levels of *GRP23* based on microarray data displayed in the eFP browser (<http://bar.utoronto.ca/efp/cgi-bin/efpWeb.cgi>). Color scale shows microarray signal level. (B) Expression levels of *GRP23* in various tissues of *Arabidopsis* detected by qRT-PCR. Data are means ± SD (*n* = 3).
