# Supplementary material for: Ectopic Expression of WUS in Hypocotyl Promotes Cell Division via GRP23 in Arabidopsis
Source: PLoS One. 2013 Sep 26;8(9):e75773. doi: 10.1371/journal.pone.0075773 (PMC3784395; doi:10.1371/journal.pone.0075773)
Supplement: Figure S4 — Expression levels of A-type ARRs genes in hypocotyl. (DOCX) [file pone.0075773.s004.docx]

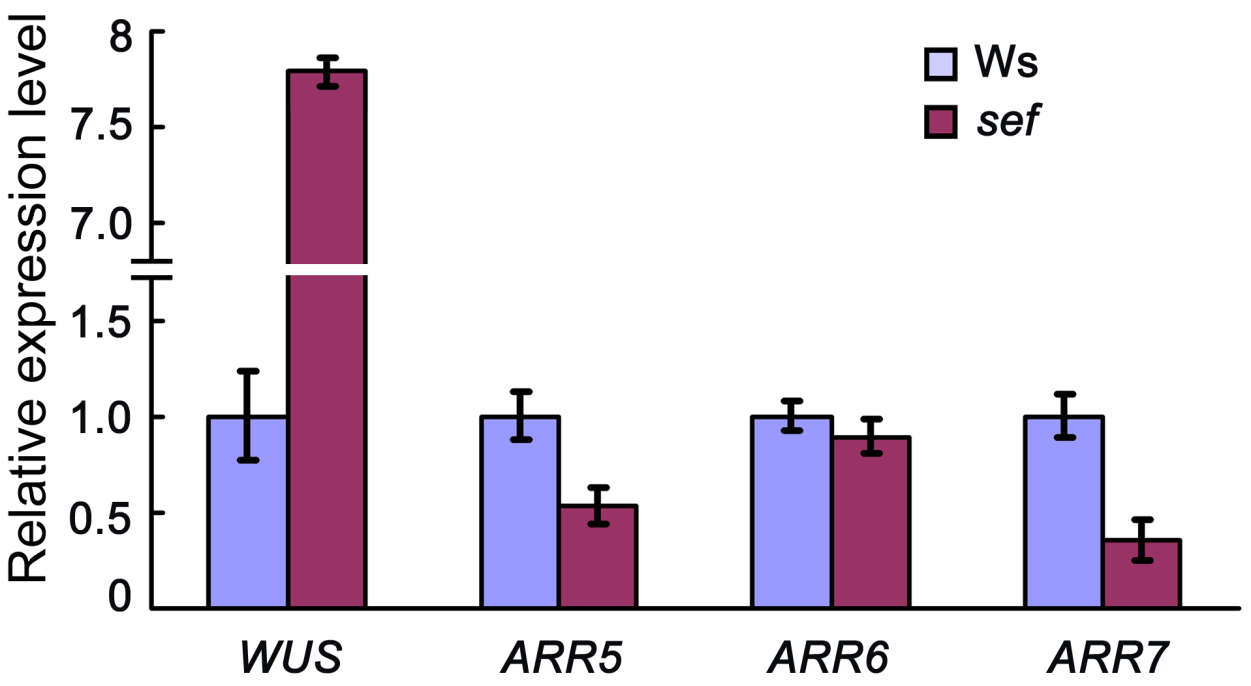


**Figure S4. Expression levels of A-type *ARRs* genes in hypocotyl**

The transcriptional levels were detected by qRT-PCR. The *Actin1* gene was used as the internal control. Data are means ± SD (*n* = 3).
